# Landraces of *temperate japonica* rice have superior alleles for improving culm strength associated with lodging resistance

**DOI:** 10.1038/s41598-020-76949-8

**Published:** 2020-11-16

**Authors:** Koki Chigira, Natsuko Kojima, Masanori Yamasaki, Kenji Yano, Shunsuke Adachi, Tomohiro Nomura, Mingjin Jiang, Keisuke Katsura, Taiichiro Ookawa

**Affiliations:** 1 Graduate School of Agriculture, Tokyo University of Agriculture and Technology, 3-5-8 Saiwai-cho, Fuchu, Tokyo 183-8509 Japan; 2 Food Resources Education and Research Center, Graduate School of Agricultural Science, Kobe University, 1348 Uzurano-cho, Kasai, Kobe, 675-2103 Japan; 3 Statistical Genetics Team, RIKEN Center for Advanced Intelligence Project, Nihonbashi, Tokyo, 103-0027 Japan; 4 College of Agriculture, Ibaraki University, 3-21-1 Chuo, Ami Town, Ibaraki 300-0393 Japan; 5 Rice Research Institute of Guizhou Academy of Agricultural Science, Guiyang, 550006 Guizhou China

**Keywords:** Natural variation in plants, Plant breeding, Plant genetics

## Abstract

Lodging can reduce grain yield and quality in cereal crops including rice (*Oryza sativa* L.). To achieve both high biomass production and lodging resistance, the breeding of new cultivars with strong culms is a promising strategy. However, little is known about the diversity of culm strength in *temperate japonica* rice and underlying genetic factors. Here, we report a wide variation of culm strength among 135 *temperate japonica* cultivars, and some landraces having the strongest culms among these cultivars. The genome-wide association study (GWAS) identified 55 quantitative trait loci for culm strength and morphological traits, and revealed several candidate genes. The superior allele of candidate gene for culm thickness, *OsRLCK191*, was found in many landraces but had not inherited to the modern improved cultivars. Our results suggest that landraces of *temperate japonica* rice have unutilized superior alleles for contributing future improvements of culm strength and lodging resistance.

## Introduction

The demand for food is increasing in developing Asian countries, which are major rice producers and consumers, due to the rapid growth of the population^1^. Moreover, Africa and Latin America are also becoming mass consumers of rice^2^, so the need to increase rice yield is a global problem.

Lodging is one of the major factors of low yield and quality in the production of rice. Once lodging occurs, the canopy photosynthetic rate decreases due to the deterioration of canopy architecture, translocation of nutrients and water is inhibited, and pre-harvest sprouting leads to poor grain quality^3,4^. To avoid lodging, the breeding programme was performed in the 1960s “Green Revolution”, aiming to introduce the semi-dwarf gene *sd1* into rice cultivars. Semi-dwarf cultivars contributed high yields because they can avoid lodging by maintaining low plant heights even in heavily fertilized conditions^5^. However, recently, the risk of super typhoon is increasing in East Asia, which is a major rice-producing area^6^. In such extreme conditions, the risk of lodging is still high even in semi-dwarf cultivars. From another aspect, high-yielding cultivars that have larger panicles with more grains were recently bred, but the culm strength of these cultivars is not enough to support their heavy panicles^7^. For these reasons, it is necessary to breed rice cultivars that have higher lodging resistance not only by semi-dwarfism but also by making their culm thicker and stiffer.

Lodging in rice is classified into three types: stem-breaking type, stem-bending type, and root lodging type. In this study, we focused on the traits associated with stem-breaking type and stem-bending type lodging, which often become a problem in transplanting cultivation. In stem-breaking type lodging resistance, the bending moment at breaking (BM) is the main indicator^8^. The BM is calculated from the section modulus (SM), which indicates the culm thickness, and the bending stress (BS), which indicates the culm stiffness. On the other hand, flexural rigidity (FR) is the indicator for stem-bending type lodging resistance, and it is calculated from the Young’s modulus (YM) and the secondary moment of inertia.

To uncover the genetic background of culm strength in rice, some quantitative trait loci (QTLs) and genes associated with culm strength have been detected. From *indica* cultivars, two QTLs, *SCM1* and *SCM2,* were detected, and the causal gene of *SCM2* was identical to *APO1*, which is associated with panicle morphogenesis^8^. From *tropical japonica* cultivars, two other QTLs, *SCM3* and *SCM4,* were detected, and the causal gene of *SCM3* was identical to *FC1/OsTB1*, which is associated with the signal pathway of strigolactone^9^. Cultivars with superior alleles of these QTLs or genes show large BM and SM, resulting high resistances to stem-breaking type lodging. On the other hand, regarding *temperate japonica* rice cultivars, only *SD1*, which is allelic to the semi-dwarf gene *sd1*, has been detected for a gene associated with culm thickness^10,11^. However, QTL analysis using chromosome segment substituted lines (CSSLs) and recombinant inbred lines (RILs) suggested that there are certain genes associated with culm strength in *temperate japonica* rice cultivars other than *SD1*^11,12^. If causal genes determining culm strength in *temperate japonica* cultivars are identified, it will be possible to improve the culm strength more efficiently. In our previous studies, as mentioned above, QTL analysis from RILs and CSSLs was used^13–15^. However, these biparental QTL analyses consider alleles from only two cultivars, so it is impossible to identify multiple genes associated with culm strength and to reveal the diversity of their alleles among a large number of cultivars at once. Recently, the progress of next-generation sequencing has enabled the acquisition of big data on DNA polymorphisms among a large number of cultivars, and genome-wide association studies (GWAS) have become a powerful tool for searching for genes^16–18^. For example, some genes associated with agronomic traits, such as heading date and plant height, have been rapidly identified from GWAS using a *temperate japonica* rice population^19,20^. Therefore, GWAS is expected to be a useful method to search for genes associated with culm strength in *temperate japonica* rice cultivars.

In this research, we used 135 cultivars of *temperate japonica* rice, including landraces established before cross breeding. Landraces of rice had been selected by farmer’s seed-raising to adapt to the local environments and have extensive variation^21^. Some of them have traits which modern improved cultivars do not have, such as very long culms. We report some important insights into the usefulness of landraces in improvement of culm strength of *temperate japonica* rice cultivars and some candidate regions of genes associated with culm strength and other agronomic traits from GWAS.

## Results

### Phenotypic diversities and relationships between traits

For this research, we cultivated 135 cultivars of *temperate japonica* rice (Table S1). To reveal the existence of natural variations on the traits associated with culm strength and the relationships between culm strength and other agronomic traits, we evaluated the phenotypic variances and the correlations of 14 quantitative traits associated with culm strength and yield. The 14 traits include 7 traits associated with culm strength: BM, BS, YM, FR, culm tissue density (CTD), and culm diameter of basal internode and third internode (BCD and 3rdCD). The other 7 traits are days to heading (DTH), culm length (CL), panicle number (PN), panicle length (PL), spikelet number per panicle (SPN), flag leaf length (FLL) and flag leaf width (FLW).

**Figure 1:**
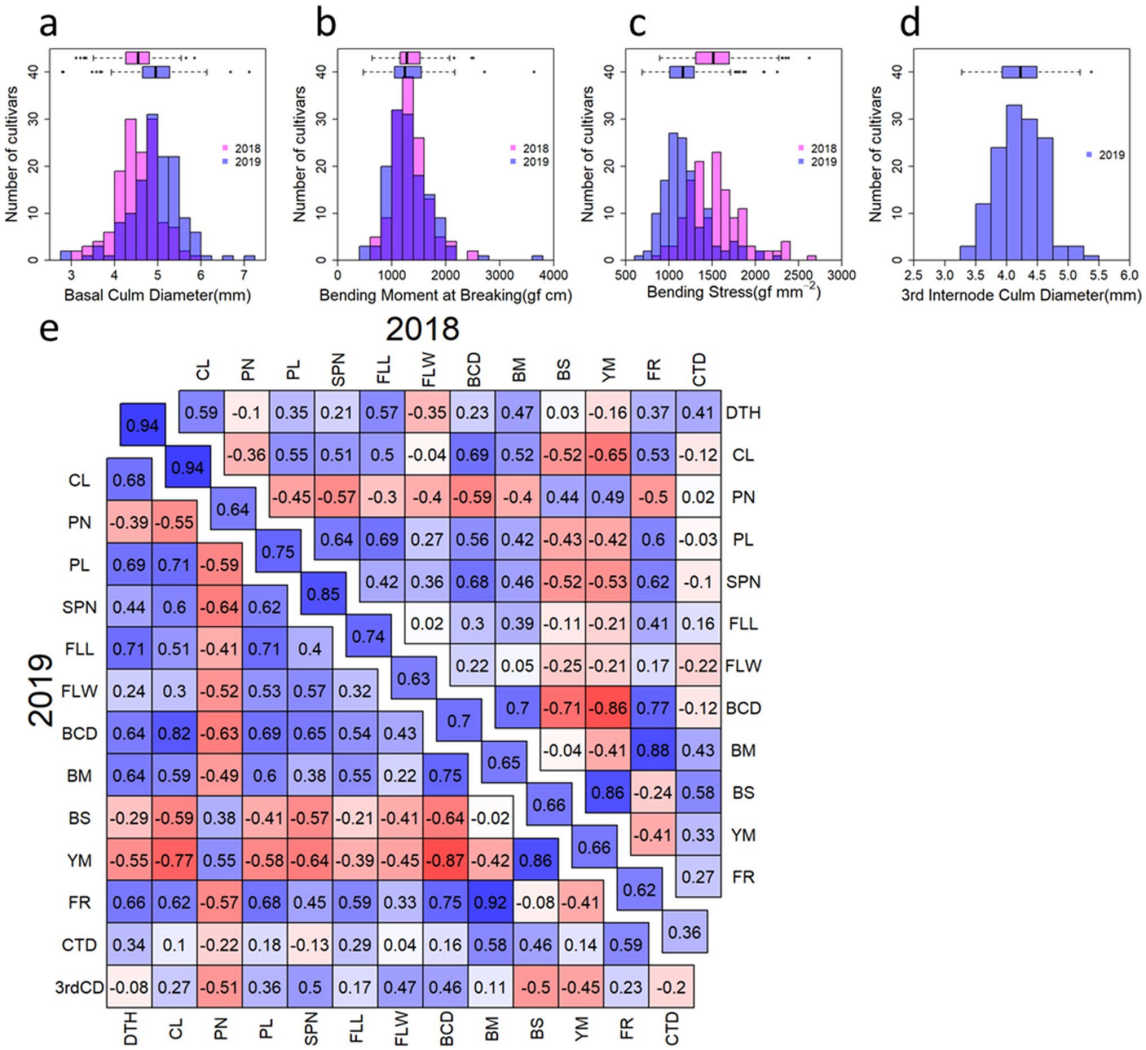
Phenotypic diversities and relationships between traits. (**a**–**d**) Histogram and boxplot of the traits associated with culm strength. Red and blue bars indicate the results in 2018 and 2019, respectively. (**a**) Basal culm diameter. (**b**) Bending moment at breaking. (**c**) Bending moment. (**d**) Culm diameter of the 3rd internode. (**e**) Correlations among 14 traits. The numbers show the correlation coefficient value (R). The number on the diagonal indicates the correlation coefficient between 2 years. The upper triangle and lower triangle show the results of 2018 and 2019, respectively.

In our results, the phenotypic variances of each trait were sufficiently large, and they were distributed (Fig. 1, Fig. S1). This indicated that the 135 *temperate japonica* rice panel contains natural variations in quantitative traits associated with culm strength and yield. Focusing on the culm traits, BM, which indicates culm strength, varied from 0.63 to 2.5 (× 10^3^ gf cm) and from 0.47 to 3.6 (× 10^3^ gf cm) in 2018 and 2019, respectively (Fig. 1b). BCD, which indicates culm thickness varied from 3.1 to 5.8 mm and from 2.8 to 7.1 mm in 2018 and 2019, respectively (Fig. 1a), and BS, which indicates culm stiffness varied from 0.89 to 2.6 (× 10^3^ gf mm^−2^) and from 0.69 to 2.3 (× 10^3^ gf mm^−2^) in 2018 and 2019, respectively (Fig. 1c). We also measured 3rdCD in 2019 because the number of elongated internodes was different among cultivars. The 3rdCD also showed a large variance (Fig. 1d).

The correlations of each trait are shown in Fig. 1e. In terms of the traits associated with culm strength, there were strong correlations among BCD, BM and BS. The correlation between 3rdCD and DTH was − 0.08, and they had low correlation, although BCD and DTH were 0.64 in 2019, and they had a positive correlation. This indicates that BCD may be affected by heading time, and 3rdCD is better to compare the culm thickness purely among cultivars.

**Figure 2:**
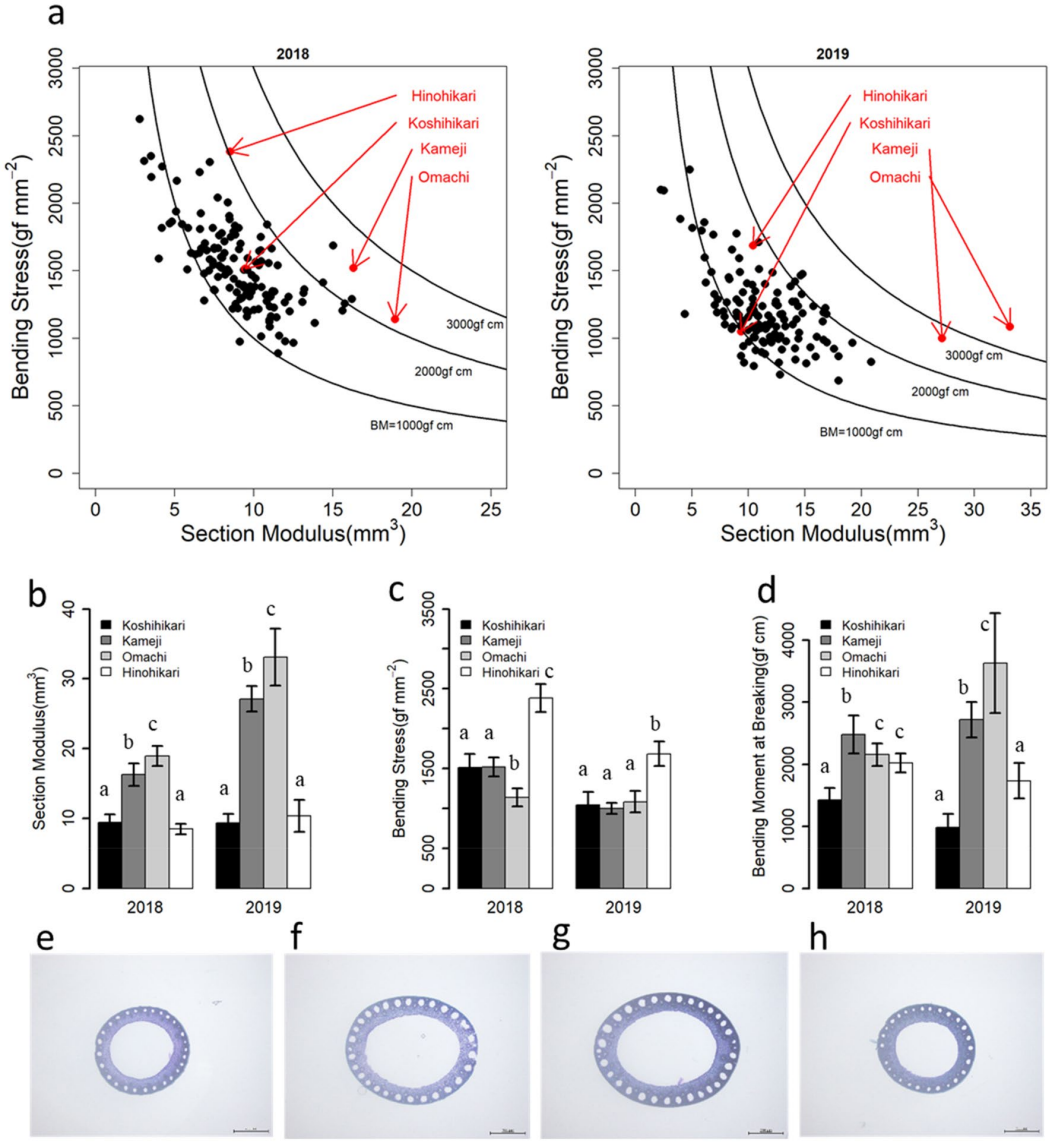
The component traits of culm strength among cultivars. (**a**) The relationships between section modulus and bending stress on 135 cultivars. The graphs on the left and right shows the results of 2018 and 2019, respectively. The curves indicate the bending moment at breaking. Red plots indicate the positions of the 4 cultivars focused on (**b**–**d**). (**b**–**d**) The section modulus, bending moment and bending moment at breaking of the 4 cultivars. Different letters indicate significant differences between both cultivars: P < 0.05 (Tukey's test). Each bar indicates the mean ± SD (n = 8, only “Hinohikari” in 2019 is n = 5). (**e**–**h**) Images of culm sections. Each picture indicates Koshihikari, Kameji, Omachi and Hinohikari, in order from the left.

To confirm the factors of the diversity of BM, comparisons of SM and BS were performed (Fig. 2a). BM is the product of SM multiplied by BS. In the 135 cultivars, there were three cultivars whose BM was considerably higher than Koshihikari, which is the most popular cultivar in Japan. Two of the three, Omachi and Kameji, had significantly higher SM than Koshihikari, although their BS values were the same as or lower than Koshihikari’s (Fig. 2b–d,e–g). On the other hand, Hinohikari had a higher BS than Koshihikari, although its SM was similar to Koshihikari’s. (Fig. 2b–d,e,h). These results suggested that there are also diversities in the component traits of culm strength among cultivars.

**Figure 3:**
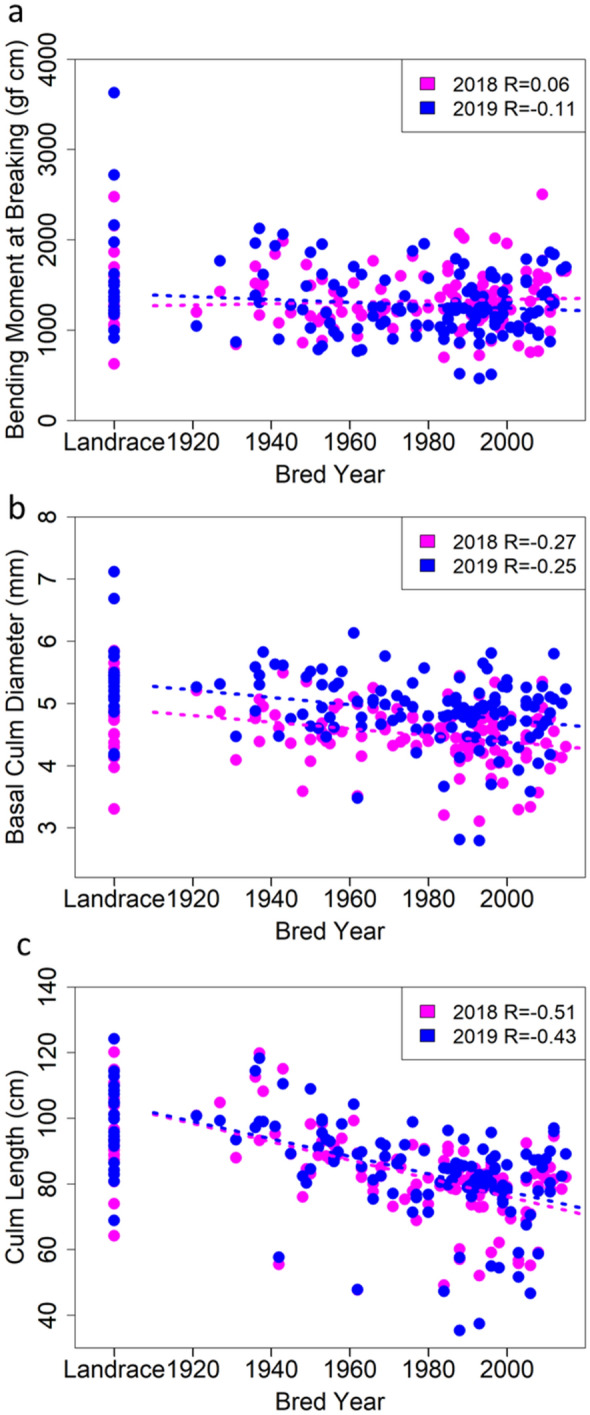
The relationships between the traits and the year each cultivar was bred. Dotted lines indicate the regression lines. Red and blue plots or lines indicate the result of 2018 and 2019, respectively. (**a**) Bending moment at breaking. (**b**) Basal culm diameter. (**c**) Culm length.

Figure 3 shows the relationships between the culm traits and the released years of cultivars. There were no significant trends of BM and BCD with the released years of cultivars (Fig. 3a,b). This suggested that less attention on the culm strength has been paid in the breeding histories. On the other hand, the CL tended to be shorter in recently grown cultivars (Fig. 3c). This indicated that the improvement of lodging resistance in Japanese *temperate japonica* rice cultivars had been carried out mainly by shortening plant height.

To confirm the contribution of culm strength to the actual lodging resistance, we investigated the degree of lodging using aerial images from an unmanned aerial vehicle (Fig. S2). The degree of lodging tended to be heavy as the CL increased, and focusing on the top 25% of cultivars in culm length, the cultivars which had the larger BM tended to have reduced degree of lodging.

### Detection of the candidate regions by GWAS

To detect the candidate regions responsible for 14 traits whose phenotypic diversities were confirmed, GWAS was performed. Before performing GWAS, to understand the genetic background of the 135 rice cultivars, principal component analysis (PCA) was performed on genotype data including 670,069 single nucleotide polymorphisms (SNPs) or insertion-deletions (InDels) (Fig. S3). Focusing on the location of the breeding programme, cultivars bred in Hokkaido (northern part of Japan) were slightly structured, but most cultivars were not highly structured. The scores of principal components 1 and 2 were 10.952% and 8.382%, respectively. This indicated that the population structure among the 135 cultivars was sufficiently small. Thus, we decided to use a linear mixed model without considering population structure for GWAS.

**Figure 4:**
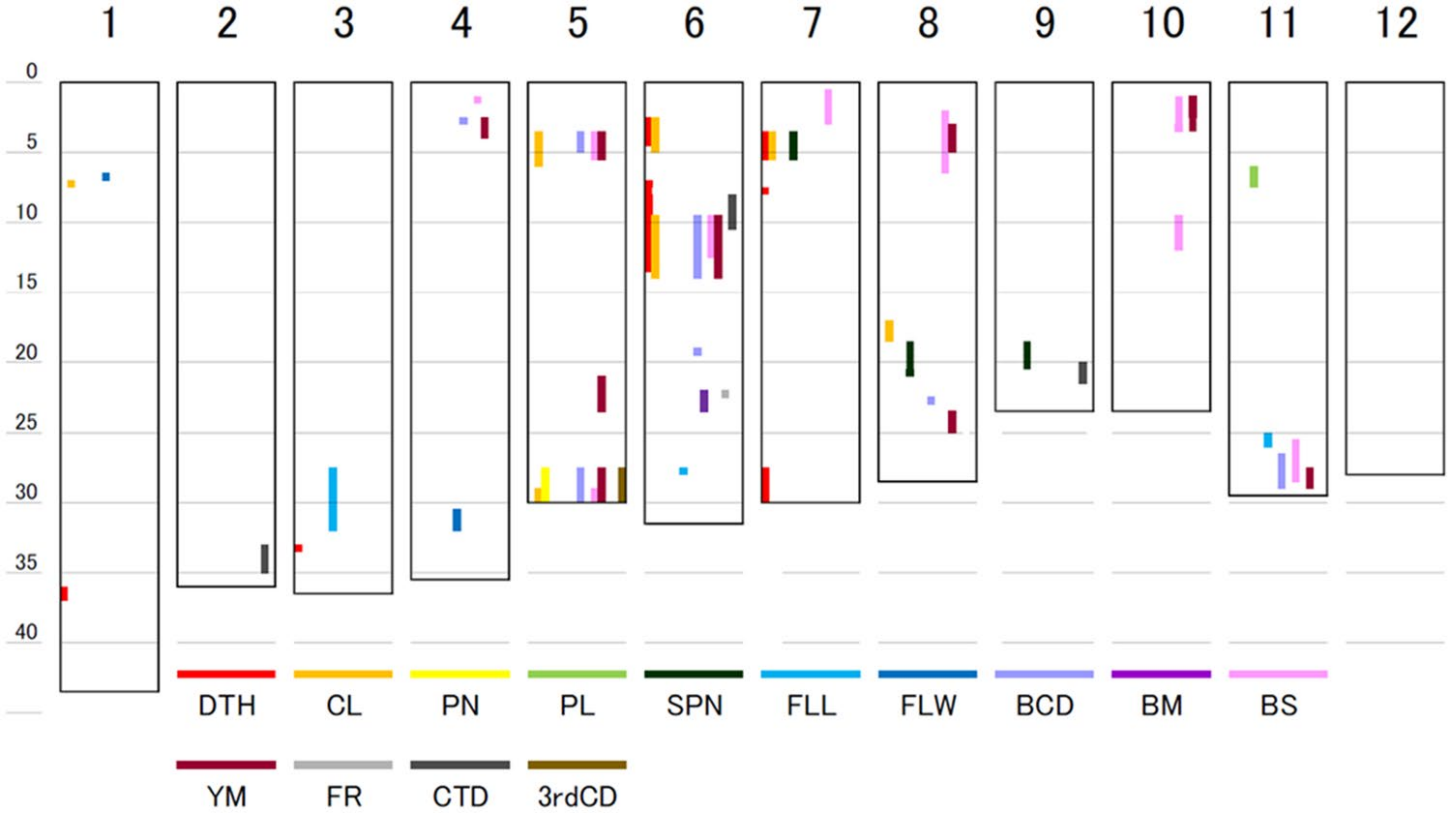
Candidate region mapping based on GWAS in 2018 and 2019. *DTH* days to heading, *CL* culm length, *PN* panicle number, *PL* panicle length, *SPN* spikelet number per panicle, *FLL* flag leaf length, *FLW* flag leaf width, *BCD* basal culm diameter, *BM* bending moment at breaking, *BS* bending stress, *YM* Young’s modulus, *FR* flexural rigidity, *CTD* culm tissue density, *3rdCD* culm diameter of 3rd internode.

We performed two types of GWAS (Fig. S4). One is DNA polymorphism-based GWAS, which analyses the association between phenotypes and 670,069 SNPs or InDels. The other is gene-based association study, which analyses the association between the phenotypes and haplotypes of 14,274 genes that had DNA mutations, such as inducing amino acid exchange. The candidate regions were defined as the chromosome regions that met two criteria: (1) DNA polymorphisms whose *p* values were under 10^–4^ in DNA polymorphism-based GWAS and (2) genes whose *p* values were under 10^–4^ in gene-based association analysis. According to these criteria, 23 and 48 candidate regions were detected in 2018 and 2019, respectively (Fig. 4, Table 1). Among them, 16 candidate regions were detected in both years. In the traits associated with culm strength, the number of candidate regions for BCD, 3rdCD, BS, BM, FR, YM and CTD were 7, 1, 1, 9, 1, 10 and 3, respectively.

**Table 1:** Candidate regions for causal genes of 14 traits.

Trait	QTL name	Year	Chr	Start	End	P value of peak	Number of significant genes	Annotated genes
Days to heading	*qDTH1*	2019	1	36,302,302	36,661,955	1.50E−05	9	*OsHESO1 (LOC_Os01g62780)*
	*qDTH3*	2018	3	33,075,629	33,082,634	5.71E−05	1	*Hd16 (LOC_Os03g57940)*
	*qDTH6-1*	2019	6	2,867,939	4,105,198	3.50E−06	1	
	*qDTH6-2*	2018	6	7,241,590	10,682,931	2.69E−09	19	*Hd1(LOC_Os06g16370)*
		2019	6	7,242,240	13,273,924	2.22E−07	14	
	*qDTH7-1*	2019	7	3,857,839	5,220,414	3.50E−06	5	
	*qDTH7-2*	2019	7	8,502,975	8,503,004	5.08E−05	1	*Ghd7 (LOC_Os07g15770)*
	*qDTH7-3*	2019	7	28,815,689	29,549,433	3.47E−06	1	*Hd2(LOC_Os07g49460)*
Culm length	*qCL1*	2019	1	7,080,206	7,239,635	1.66E−05	1	
	*qCL5-1*	2018	5	3,796,606	5,731,804	1.21E−05	1	
		2019	5	3,796,606	4,504,388	1.69E−07	1	
	*qCL5-2*	2019	5	29,126,924	29,928,598	3.41E−05	18	
	*qCL6-1*	2018	6	2,867,939	4,564,993	8.08E−07	20	
		2019	6	2,867,939	4,564,993	3.06E−06	14	
	*qCL6-2*	2018	6	13,137,626	13,573,334	1.34E−05	2	
		2019	6	9,907,766	13,752,138	1.22E−06	15	
	*qCL7*	2018	7	3,857,839	5,462,601	1.01E−05	4	
		2019	7	3,791,728	5,462,601	5.69E−06	4	
	*qCL8*	2018	8	17,002,605	18,113,848	1.54E−05	2	
		2019	8	18,113,848	18,113,848	7.75E−05	2	
Panicle number	*qPN5*	2018	5	27,940,991	29,363,499	9.42E−07	4	
Panicle length	*qPL11*	2019	11	6,369,017	7,194,273	1.72E−05	1	*sp1(LOC_Os11g12740)*
Spikelet number	*qSPN7*	2019	7	3,855,482	5,222,866	5.73E−06	4	
	*qSPN8*	2018	8	18,739,837	20,501,113	1.37E−05	1	
	*qSPN9*	2019	9	18,970,742	20,488,878	4.35E−05	10	
Flag leaf length	*qFLL3*	2018	3	27,926,299	30,995,908	1.86E−08	9	
		2019	3	27,929,416	31,512,460	5.78E−06	4	*Hd6(LOC_Os03g55389)*
	*qFLL6*	2018	6	27,217,182	27,418,688	4.26E−05	2	
	*qFLL11*	2018	11	25,450,620	25,503,471	5.07E−05	2	
Flag leaf width	*qFLW1*	2018	1	6,618,896	6,724,386	2.87E−05	1	
		2019	1	6,618,896	6,647,986	1.51E−05	1	
	*qFLW4*	2018	4	30,746,467	31,502,547	7.00E−07	11	*NAL1 (LOC_Os04g52479)*
		2019	4	31,157,451	31,352,197	3.00E−05	6	
Basal culm diameter	*qBCD4*	2019	4	2,691,755	2,691,755	1.02E−05	2	
	*qBCD5-1*	2019	5	3,796,606	4,504,388	1.94E−06	1	
	*qBCD5-2*	2018	5	27,940,991	29,890,589	7.86E−06	4	
		2019	5	27,505,030	29,928,598	3.39E−07	20	
	*qBCD6-1*	2019	6	9,907,766	13,752,138	6.32E−07	14	
	*qBCD6-2*	2019	6	19,093,132	19,093,132	7.76E−05	2	
	*qBCD8*	2019	8	23,677,530	23,962,828	3.55E−05	2	
	*qBCD11*	2019	11	27,724,930	29,015,100	3.49E−05	19	
Breaking moment	*qBM6*	2019	6	22,823,316	23,019,186	6.47E−07	1	
Bending stress	*qBS4*	2019	4	1,283,779	1,283,779	2.56E−06	1	
	*qBS5-1*	2019	5	3,796,606	5,463,605	3.13E−07	6	
	*qBS5-2*	2019	5	29,126,924	29,928,598	4.29E−06	19	
	*qBS6*	2018	6	9,925,796	10,075,483	3.64E−06	3	
		2019	6	9,907,766	12,107,939	4.68E−07	12	
	*qBS7*	2018	7	757,788	2,705,676	6.98E−06	1	
	*qBS8*	2019	8	2,143,950	6,043,554	4.29E−07	2	
	*qBS10-1*	2019	10	1,113,943	3,297,222	3.90E−08	4	
	*qBS10-2*	2019	10	9,718,000	11,691,675	1.90E−07	1	
	*qBS11*	2018	11	27,812,322	27,828,581	4.54E−05	1	
		2019	11	25,790,142	28,446,869	1.85E−05	19	
Young’s modulus	*qYM4*	2019	4	2,840,514	3,546,128	2.03E−05	3	
	*qYM5-1*	2018	5	3,796,606	5,312,775	2.05E−05	1	
		2019	5	3,796,606	5,312,775	3.43E−08	2	
	*qYM5-2*	2018	5	21,334,298	23,321,036	5.10E−06	2	
		2019	5	22,307,597	23,321,036	2.23E−06	1	
	*qYM5-3*	2018	5	29,126,924	29,928,598	6.01E−05	4	
		2019	5	27,398,408	29,928,598	1.88E−08	20	
	*qYM6*	2019	6	9,907,766	13,752,138	3.56E−07	17	
	*qYM8-1*	2019	8	3,469,204	4,596,332	3.95E−07	3	
	*qYM8-2*	2019	8	23,677,530	24,784,308	1.76E−07	3	
	*qYM10*	2019	10	1,113,943	3,297,222	3.02E−07	4	
	*qYM11*	2018	11	27,670,642	28,857,992	1.24E−07	4	
		2019	11	27,724,930	29,015,100	9.78E−07	45	
Flexural rigidity	*qFR6*	2019	6	22,823,316	22,994,705	3.13E−06	1	
Culm tissue density	*qCTD2*	2019	2	33,208,146	34,879,419	5.80E−06	2	
	*qCTD6*	2018	6	8,197,078	10,452,678	1.69E−05	5	
	*qCTD9*	2019	9	20,466,533	21,189,653	5.61E−05	8	
Culm diameter (3rd)	*q3CD5*	2019	5	27,505,030	29,682,002	8.40E−08	11	

For the traits associated with culm strength, some candidate regions were detected at the same position for multiple traits (Fig. 4). Candidate regions of CL, BCD, BS and YM were located on the short arm of chromosome 5, from approximately 3–6 Mb. Similarly, candidate regions of CL, PN, BCD, 3rdCD, BS and YM were positioned at the terminal end of the long arm of chromosome 5, from approximately 27 Mb to the terminus. Furthermore, candidate regions of DTH, CL, BCD, BS, YM and CTD were positioned at the short arm of chromosome 6, from approximately 7–13 Mb. Candidate regions of BCD, BS, and YM were positioned at the terminal end of the short arm of chromosome 11, from approximately 26 Mb to terminal.

### Detailed search for candidate genes

**Figure 5:**
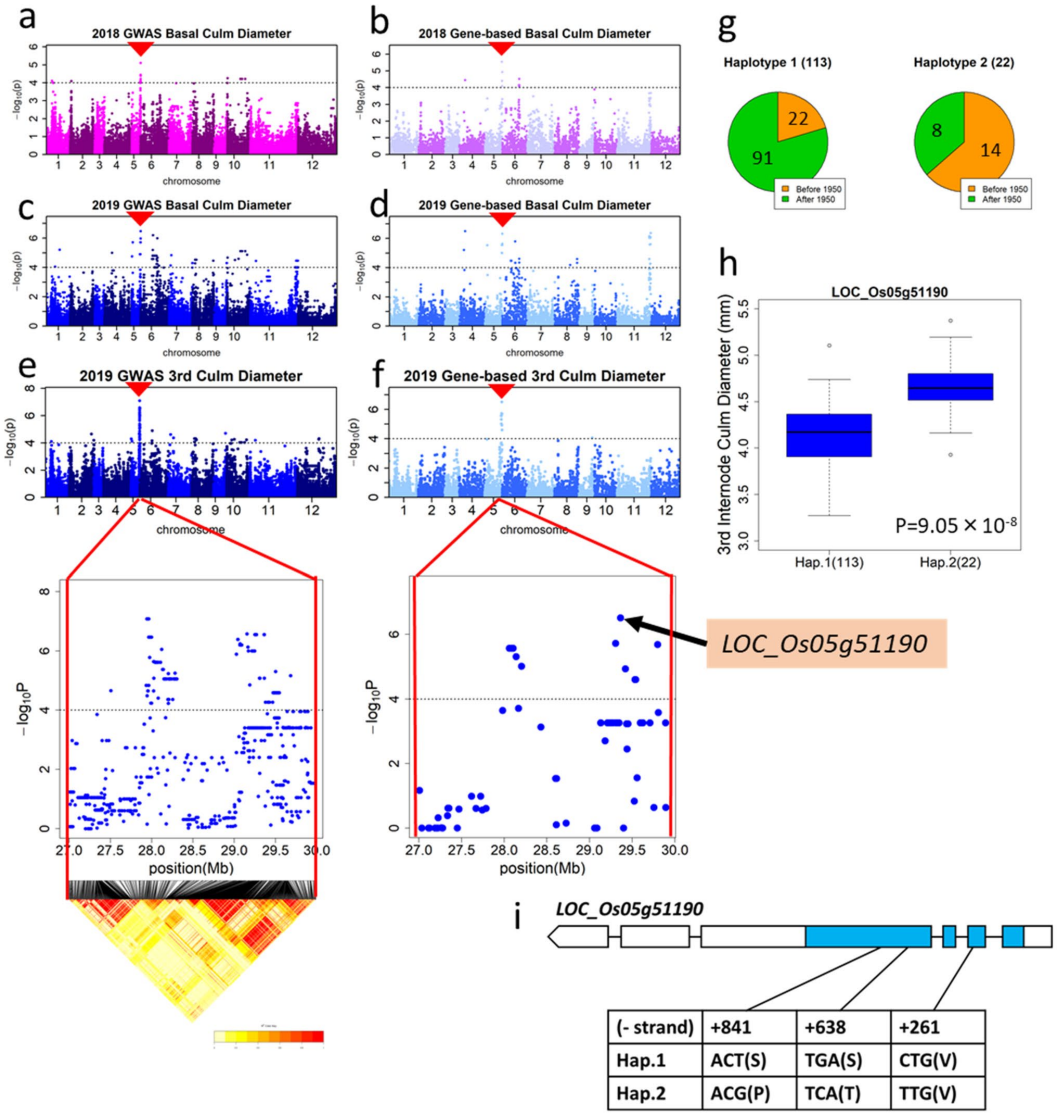
The candidate region detected between 27 Mb and the terminal end of chromosome 5. (**a**) The Manhattan plot of GWAS for basal culm diameter in 2018. The red triangle indicates the candidate region on chromosome 5, and the same applies hereinafter. (**b**) The Manhattan plot of the gene-based association study for basal culm diameter in 2018. (**c**) The Manhattan plot of GWAS for basal culm diameter in 2019. (**d**) The Manhattan plot of the gene-based association study for basal culm diameter in 2019. (**e**) The Manhattan plot of GWAS for the culm diameter of the 3rd internode in 2019. The figure below is the enlarged view of the candidate region. (**f**) The Manhattan plot of the gene-based association study for the culm diameter of the 3rd internode in 2019. The figure below is the enlarged view of the candidate region. (**g**) The ratio of the cultivars bred before 1950 and after 1950 in each haplotype of *LOC_Os05g51190* (1950 is included after 1950). The numbers on the graph indicate the numbers of cultivars included. (**h**) The culm diameter of the 3rd internode for the indicated haplotype of *LOC_Os05g51190*. Differences between the haplotypes were analysed by Welch’s t-test. (**i**) The DNA mutations in the coding region of *LOC_Os05g51190*.

In the Manhattan plot, which shows the GWAS results, the highest peak was observed at the candidate region positioned between 27 Mb and the terminus of chromosome 5 for BCD, 3rdCD, and PN (Fig. 5a,c,e, Fig. S5). We determined this region to be the most likely candidate region because, as mentioned above, 3rdCD indicated the culm thickness independently from the heading date, and this was the only region detected for 3rdCD.

We made a local Manhattan plot of the candidate region to obtain more detailed information on the results of the GWAS and gene-based association study (Fig. 5b,d,e,f, Fig. S5). Then, we defined candidate responsible genes as genes whose *p* value in the gene-based association study was under 10^–4^. In BCD and 3rdCD, there were 21 and 11 candidate genes, respectively (Fig. 5f, Tables S3, S4). BCD and 3rdCD had four common candidate genes. In particular, *LOC_Os05g51190* had the smallest *p* value. *LOC_Os05g51190* had two haplotypes in our *temperate japonica* rice population. Haplotype 1 contains 113 cultivars, including the major improved cultivars such as “Nipponbare” and “Koshihikari”. On the other hand, haplotype 2 contains 22 cultivars, including many landraces such as “Omachi” (Fig. 5g, Table S4). The values of 3rdCD were significantly larger in the cultivars with haplotype 2 than in those with haplotype 1 (P = 9.05 × 10^–8^), although the value of PN was smaller in haplotype 2 (P = 7.25 × 10^–6^ in 2018, 2.35 × 10^–5^ in 2019) (Fig. 5h, Fig. S6). Haplotype 1 contained the same sequences as the reference genome, and haplotype 2 contained three polymorphisms, including two candidate variants that cause amino acid substitution mutations (Fig. 5i).

**Figure 6:**
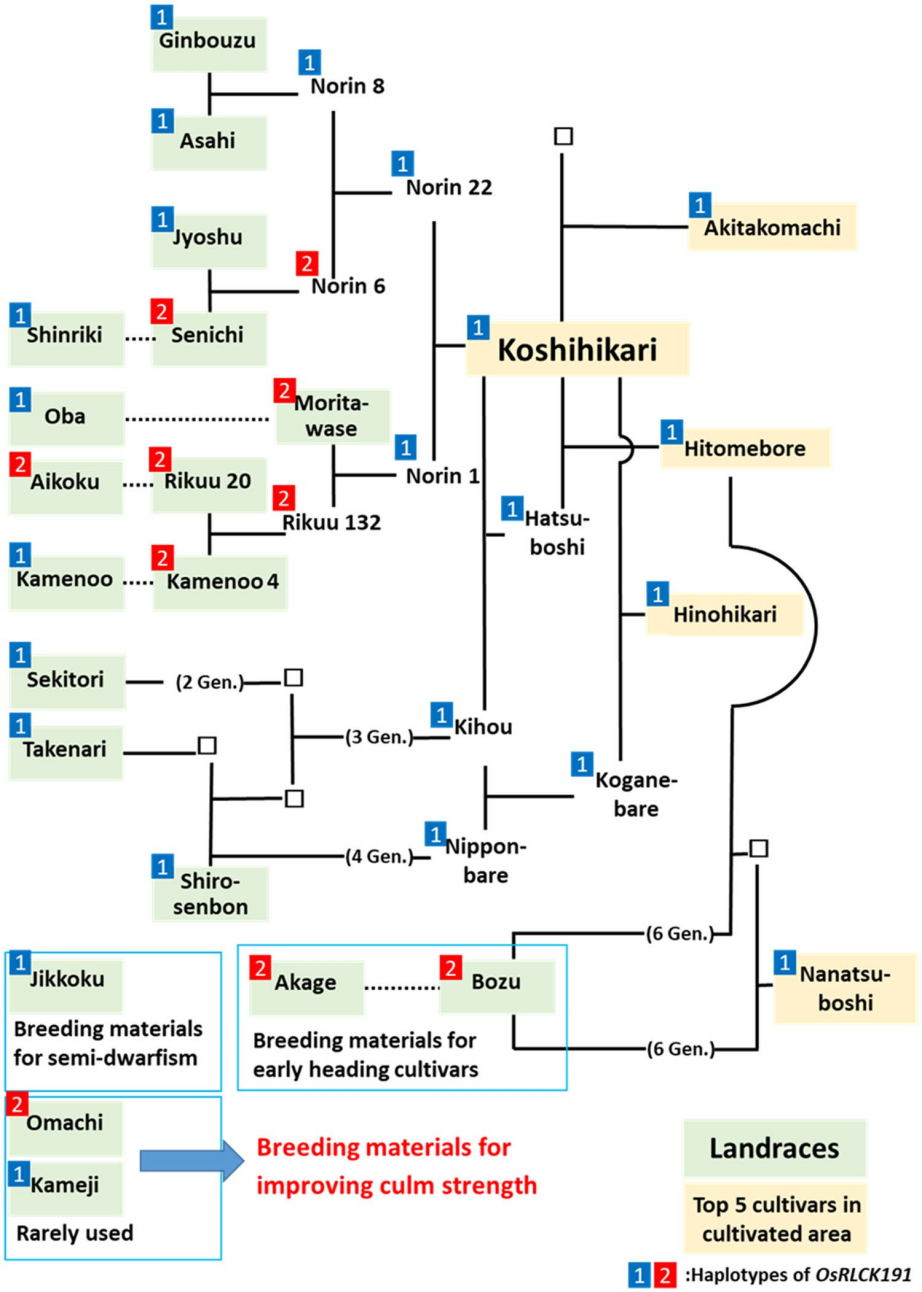
The breeding history of the top 5 cultivars in the cultivation area in Japan and their relationships with landraces.

To visualize which haplotype was inherited through modern breeding, the breeding history of the top 5 improved cultivars of cultivation area in Japan is shown in Fig. 6. Haplotype 2 was not inherited to any of these leading cultivars, although the 7 landraces which are ancestor of these leading cultivars had Haplotype 2. These results indicated that there were some superior alleles that contributed to strong culms in *temperate japonica* cultivars, especially in landraces, but they have not been used in modern breeding.

## Discussion

### The diversity of culm traits in *temperate japonica* rice

In a previous study, Ookawa and Ishihara^22^ investigated these traits using 22 rice cultivars, including Japanese *temperate japonica*, *indica* and *tropical japonica* cultivars, and reported that there was little difference in BM among *temperate japonica* cultivars because the negative correlation between SM and BS was strong. However, in this study, we revealed the diversities of the traits associated with culm strength using 135 *temperate japonica* rice cultivars. Furthermore, we found two outstanding landraces with strong culms, “Omachi” and “Kameji”, which had significantly large SMs while maintaining the same BS as “Koshihikari” (Fig. 2). This result suggested that there were unknown responsible genes associated with culm strength in *temperate japonica* rice, and some landraces had superior alleles which are useful for improvement of culm thickness.

### Possibility of improving lodging resistance using landraces

Our results indicated that the improvement of lodging resistance in Japanese *temperate japonica* rice cultivars had been carried out by shortening plant height only, and the culm strength had not been improved (Fig. 3). However, the lodging resistance of “Koshihikari”, which is the leading cultivar in Japan, is low because it has long culms^23^. Moreover, most cultivars bred from "Koshihikari" are genetically close each other and have little improvement in lodging resistance^24^, so even improvement of lodging resistance by shortening plant height is not sufficient.

According to the kinships between the 20 landraces used in this study and the top 5 improved cultivars of cultivation area in Japan, the 17 landraces out of the 20 were the ancestor of those improved cultivars (Fig. 6). This indicates that Japanese improved rice cultivars have been bred with low genetic diversity. The other 3 landraces contain “Jikkoku”, “Omachi”, and “Kameji”. “Jikkoku” is the cultivar with the Japanese native semi-dwarf allele of *sd1*^25^ and is used for breeding semi-dwarf cultivars such as “Houyoku” and “Reiho”. In contrast, “Omachi” and “Kameji” have rarely been used for breeding modern good taste cultivars in Japan, although they were the top two cultivars in culm strength in our study.

“Omachi” has traits suitable for sake brewing (large and white core grain), and has been used for breeding material of cultivars for sake brewing. In our previous study, *SCM2*/*APO1*, a gene which controls the number of cells in shoot apical meristems, has the effect of simultaneously increasing the number of spikelets per panicle and the thickness of stems^8^. It was also reported that the line with an allele that increases the 1000-grain weight also increased the culm diameter and contributed to culm strength^26^. Considering from these studies, the thick culm of “Omachi” might also be related to its large grain size and its inherent traits suitable for brewing. The other landrace, “Kameji” was used as an important breeding material in Taiwan^27^. Previous study suggested that Kameji was genetically close to *indica* rice cultivars^28^, so it might have been introduced to Southeast Asia as a breeding material for adapting to subtropical areas.

No report was found that the strong culm of "Omachi" and "Kameji" were used for modern breeding. This is probably because these varieties have long stems and the actual lodging resistance is not enough. In fact, lodging resistance is a complex trait and is affected by various factors such as plant height, conditions of cultivation, and weather, so the correlation between culm strength and the degree of lodging is not always high. However, genetic factors behind complex traits can only be clarified and utilized by studying each of the component traits that constitute the complex trait. In future breeding, it is possible to improve the lodging resistance of Japanese *temperate japonica* rice cultivars by introducing the superior alleles for component traits associated with culm strength from these two landraces into the good taste cultivars.

### Relationships with QTL identified in previous studies

Regarding component traits associated with culm strength, the clearest peak of the Manhattan plot was detected at the terminal end of the longer arm of chromosome 5 for the 3rdCD (Fig. 5). This candidate region was detected not only on 3rdCD but also on the PN in 2018 (Fig. S5). This suggests that there is a gene associated with both plant architecture and culm thickness in the candidate region. In a previous study, the same region was detected as the QTL for grain width and one panicle weight from CSSLs of “Yamadanishiki”, which is a cultivar for sake brewing, and “Koshihikari”^29,30^. The genes for culm strength identified in a previous study had pleiotropic effects on panicle morphogenesis^8,9^, so it is considered that the gene responsible for this candidate region has a pleiotropic effect in the same way.

In other QTLs associated with culm strength, *qBCD5-1* (*qBS5-1*, *qYM5-1*) on the short arm of chromosome 5, *qBCD6-1* (*qBS6*, *qYM6*) on chromosome 6, and *qBCD11* (*qBS11*, *qYM11*) on chromosome 11 were detected in multiple traits, respectively (Table 1). Of these, *qBCD6-1* is located in the region where the *Heading date 1* (*Hd1*) gene exists^31^. Considering that BCD, BS, YM had relatively high correlations with DTH, it is considered that this QTL is likely to have been influenced by *Hd1*. In the regions of other two QTLs, *qBCD5-1* and *qBCD11*, we could not find known genes likely to be related to culm strength. These QTLs may contain novel genes related to culm strength. However, the same regions were not detected in 3rdCD, which had a low correlation with DTH, so there is also a possibility of false positives due to the influence of heading date.

### A candidate gene and its haplotypes derived from landraces

We have concluded that the most likely candidate gene associated with culm thickness in the candidate region is *LOC_Os05g51190* (*OsRLCK191*), which has two haplotypes. As Fig. 6 shows, Haplotype 2 seems to have been culled in the breeding history and is not used in modern commercial cultivars in Japan. This further supports the hypothesis that landraces have superior alleles which are useful to improve culm strength. On the other hand, it is needed to expand the scope of research to the cultivars outside Japan because landraces with haplotype 2 have been used outside Japan, for example, “Omachi” was used as a breeding material of Californian rice cultivars^32^. It was also interesting that “Kameji”, which had very thick culm had haplotype 1. This suggests the existence of another gene and valuable allele associated with culm thickness.

The candidate gene, *OsRLCK191* codes receptor-like cytoplasmic kinase (RLCK). In rice, over 300 RLCKs have been identified^33^. RLCK belongs to the receptor-like kinase (RLK) protein family, which is one of the largest protein families in plants. It has been reported that RLK is involved in various physiological functions of higher plants, such as the response to hormones, cell differentiation, growth, development, responses to environmental stresses, and pathogen recognition^34,35^. At present, the functions directly associated with culm thickness have not been reported for RLCK. However, it has been reported that some RLCKs that work as negative regulators of brassinosteroid signalling were associated with a decrease in panicle number when they were silenced by RNAi^36^. Our previous study showed that there was a correlation between tiller number and culm thickness, so RLCK can also be associated with culm thickness. To date, no physiological functions have been investigated for the candidate gene *OsRLCK191*. We need to research this candidate gene in more detail using gene expression analysis and evaluation of transgenic plants.

In conclusion, QTLs and candidate genes identified in this study are expected to be important information for the progress of research on culm strength and morphogenesis in rice. The fact that there are superior alleles for culm thickness in landraces suggests that rice breeding using landraces may be effective to improve lodging resistance, in addition to cross breeding between improved varieties.

## Methods

### Plant materials and cultivation

We used 135 *temperate japonica* rice cultivars, which have been cultivated with a certain share in Japan or used as parents of popular cultivars (Table S1). Field experiments were carried out at the Experimental Farm of Field Science Center in Fuchu-Honmachi, Tokyo University of Agriculture and Technology, in 2018 and 2019. Seedlings were transplanted to alluvial soil of the Tama River at a rate of one plant per hill. The density of plants was 22.2 hills per m^2^ (each space was 30 cm × 15 cm). Fifty plants for each cultivar were cultivated as one repetition. N, P_2_O_5_ and K_2_O were applied at 50 kg ha^−1^, 60 kg ha^−1^ and 60 kg ha^−1^, respectively, as a basal dressing.

### Phenotyping

We recorded the heading date of the main culms for each cultivar and sampled the main culms 15 days after heading. Then, we counted their panicle number. The 6–8 main culms that had an average length of the basal internode were chosen and used for phenotyping. The basal internode was defined as the internode positioned at the bottom of the culms but had a length over 4 cm. Morphological traits, including culm length, panicle length, spikelet number per panicle, flag leaf length and flag leaf width, were measured for each sample. For the physical parameters associated with culm strength, we recorded the bending moment at breaking and the relationships between the load and deflection of basal internodes using a Tensilon RTG-1210 universal testing machine (A&D, Tokyo, Japan). We also measured the culm thickness of basal internodes by assuming the section to be an ellipse with a hollow shape. In addition, the culm thickness of the 3rd internode was measured in the same way in 2019. From these parameters, the values of traits associated with culm strength were calculated using the following formula, based on the method of Ookawa and Ishihara^22^.

1 $$ M = \sigma Z $$

2 $$ Z = \frac{{\pi \left( {a_{1}^{3} b_{1} - a_{2}^{3} b_{2} } \right)}}{{32a_{1} }} $$

3 $$ EI = \frac{{Wl^{3} }}{\delta } $$

4 $$ I = \frac{{\pi \left( {a_{1}^{3} b_{1} - a_{2}^{3} b_{2} } \right)}}{64} $$

(1) $$M$$ is the bending moment at breaking, $$\sigma$$ is the bending stress, and $$Z$$ is the section modulus. (2) $$a_{1}$$ is the outer diameter of the minor axis, $$b_{1}$$ is the outer diameter of the major axis, $$a_{2}$$ is the inner diameter of the minor axis, and $$b_{2}$$ is the inner diameter of the major axis. (3) $$EI$$ is the flexural rigidity, $$W$$ is the load, $$l$$ is the fulcrum distance, $$\delta$$ is the deflection, (4)$$ I$$ is the secondary moment of inertia, and $$E$$ is Young’s modulus.

After the measurement, the dry weight of the basal internodes was recorded, and the culm tissue density was calculated by the following formula.

5 $$ D = \frac{{W_{d} }}{{\pi \left( {a_{1} b_{1} - a_{2} b_{2} } \right)}} . $$

(5) $D$ is the culm tissue density, and $W_{d}$ is the dry weight of the basal internodes.

The degree of lodging was determined for each variety using aerial photoaphs taken by a Mavic 2 Pro (DJI) about 30 days after heading. 3D models of the field was created using the Pix4Dcloud (https://cloud.pix4d.com/), referring to the method of Su et al.^37^. The degree of lodging was evaluated on a scale of 0–4 based on the perspective.

### Genotyping

The DNA sequences of each cultivar were analysed by an Illumina HiSeq 2000/2500 system (Illumina Co, Ltd.)^38,39^ and mapped to Os-Nipponbare-Reference-IRGSP-1.0^40,41^. We identified a total of 670,069 single nucleotide polymorphisms (SNPs) and InDels after removing nucleotide variations with missing rates > 0.10 and a minor allele frequency < 0.025. To illustrate the population structure from genotype data, we performed principal component analysis using the R function “prcomp”.

### GWAS

For GWAS, we used a linear mixed model^42^, following the method of Yano et al. (2016). We performed GWAS using the R package “rrBLUP”^43^ with the following model.

6 $$ y = X\beta + Zu + \varepsilon $$

(6) $y$ is the vector of phenotypes, $X$ is the matrix of DNA polymorphisms, $\beta$ is the vector of assumed fixed effects by DNA polymorphism, $Z$ is the incidence matrix between $y$ and $u$, and $u$ is the random effects by genetic background. $u$ was assumed by N(0, $K\sigma^{2}_{G}$), where $K$ is the kinship matrix, and $\sigma^{2}_{G}$ is the genetic variance.$\varepsilon$ is the matrix of residual effects and was assumed by N(0, $I\sigma^{2}_{E}$), where $I$ is an identity matrix, and $\sigma^{2}_{E}$ is the residual variance.

To perform a gene-based association study, we extracted the DNA mutations that cause mutations in the coding region using SNPEff^44^. Then, we identified the haplotypes of 14,274 genes, where the important DNA mutation boards. We performed a gene-based association study by substituting $X$ in the model of (6) with the haplotype data using the R script used in Yano et al.^19^.

The linkage disequilibrium (LD) heatmaps around peaks in the GWAS were illustrated using the R package “LDheatmap”^45^.

### Detection of the candidate regions and genes

We defined the candidate regions as chromosome regions that met the following two criteria: (1) DNA polymorphisms whose *p* value was under 10^–4^ in GWAS and (2) genes whose *p* value was under 10^–4^ in gene-based association analysis. We also defined the genes in the candidate region whose *p* value in the gene-based association study was under 10^–4^ as candidate genes. The annotation information of the candidate genes was downloaded from RAP-DB (https://rapdb.dna.affrc.go.jp/).

## Supplementary information

Supplementary Information

## Abbreviations

3rdCD: Culm diameter of 3rd internode
BCD: Basal culm diameter
BM: Bending moment at breaking
BS: Bending stress
CL: Culm length
CTD: Culm tissue density
DTH: Days to heading
FR: Flexural rigidity
GWAS: Genome-wide association study
PCA: Principal component analysis
PL: Panicle length
PN: Panicle number
QTL: Quantitative trait loci
SNP: Single nucleotide polymorphism
YM: Young’s modulus
