# Supplementary material for: Landraces of temperate japonica rice have superior alleles for improving culm strength associated with lodging resistance
Source: Sci Rep. 2020 Nov 16;10:19855. doi: 10.1038/s41598-020-76949-8 (PMC7670413; doi:10.1038/s41598-020-76949-8)
Supplement: Supplementary file 1 — Supplementary Information [file 41598_2020_76949_MOESM1_ESM.pdf]

Supplementary Information

Landraces of temperate japonica rice have superior alleles for improving culm strength associated with lodging resistance

Koki Chigira[1)], Natsuko Kojima[1)], Masanori Yamasaki[2)], Kenji Yano[3)], Shunsuke Adachi[4)], Tomohiro Nomura[1)], Mingjin Jiang[5)], Keisuke Katsura[1)] and Taiichiro Ookawa[1)*]

[1)]Graduate School of Agriculture, Tokyo University of Agriculture and Technology, 3-5-8 Saiwai-cho, Fuchu, Tokyo 183-8509, Japan; [2)] Food Resources Education and Research Center, Graduate School of Agricultural Science, Kobe University, 1348 Uzurano-cho, Kasai, Kobe 675-2103, Japan; [3)] Statistical Genetics Team, RIKEN Center for Advanced Intelligence Project, Nihonbashi, 103-0027 Tokyo, Japan; [4)] College of Agriculture, Ibaraki University, 3-21-1 Chuo, Ami town, Ibaraki 300-0393, Japan; [5)]Rice Research Institute of Guizhou Academy of Agricultural Science, Guiyang, Guizhou, 550006, China.

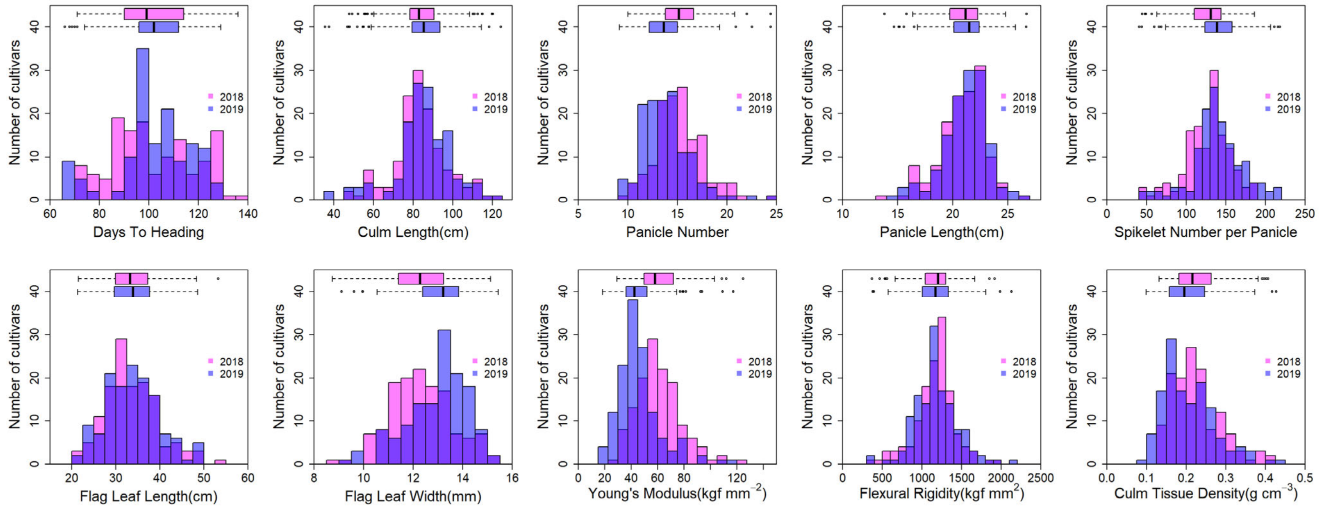

Figure S1. Histogram and boxplot of the traits that are not shown in Fig. 1.
The names of traits are shown below each histogram. Red and blue bars indicate the results in 2018 and 2019, respectively.

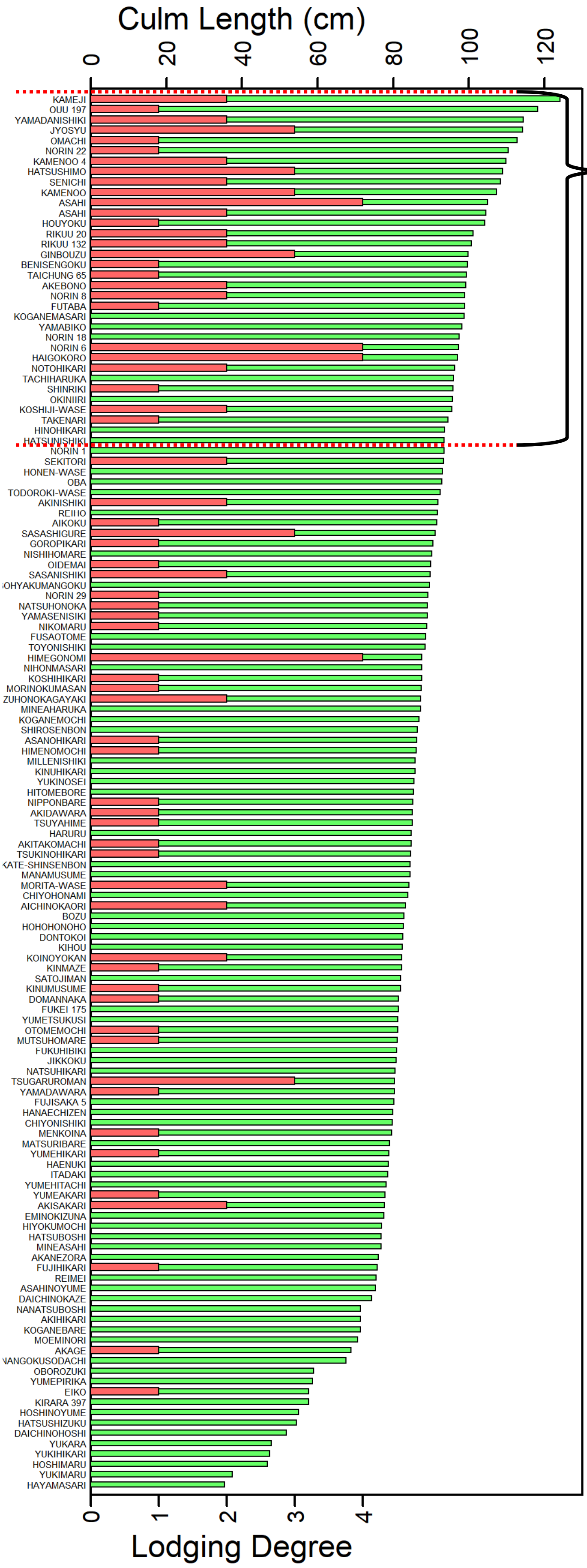

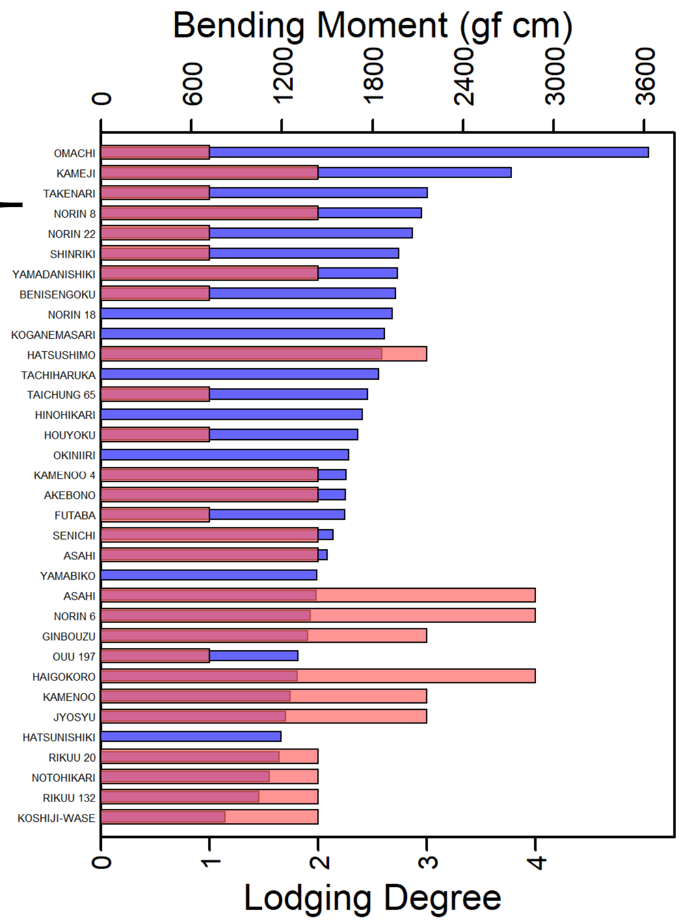

Figure S2. (Left) The degree of lodging and culm length of 135 cultivars. Cultivars are ordered by culm length. Green and red bars indicate culm length and lodging degree, respectively. (Above) the relationship between lodging degree and bending moment in the top 25% cultivars in culm length. Brue and red bars indicate bending moment and lodging degree, respectively.

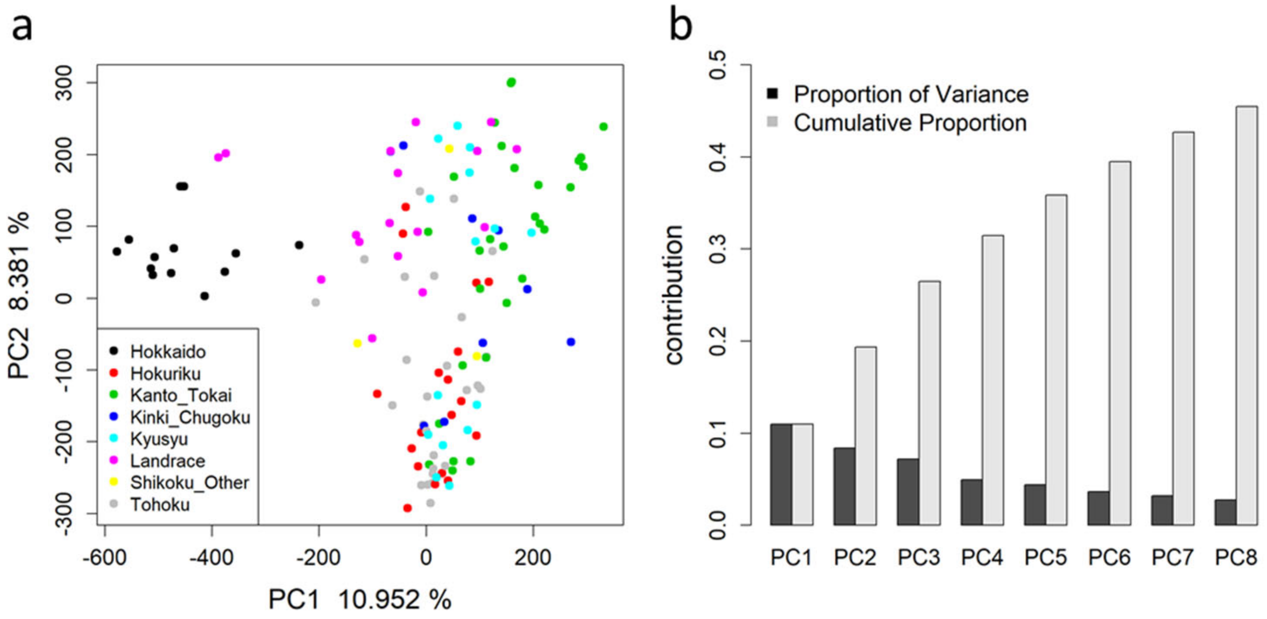

Figure S3. Population structure of the 135 cultivars.
(a)Principal component analysis for 135 cultivars using 670,069 SNPs or indels data. Percentages on labels indicate proportion of variance. Each color indicates roots of cultivars in Japan.
(b)proportion of variance of PC1 to PC8. Black bar indicates contribution of each principal components, gray bar indicates cumulative proportion.

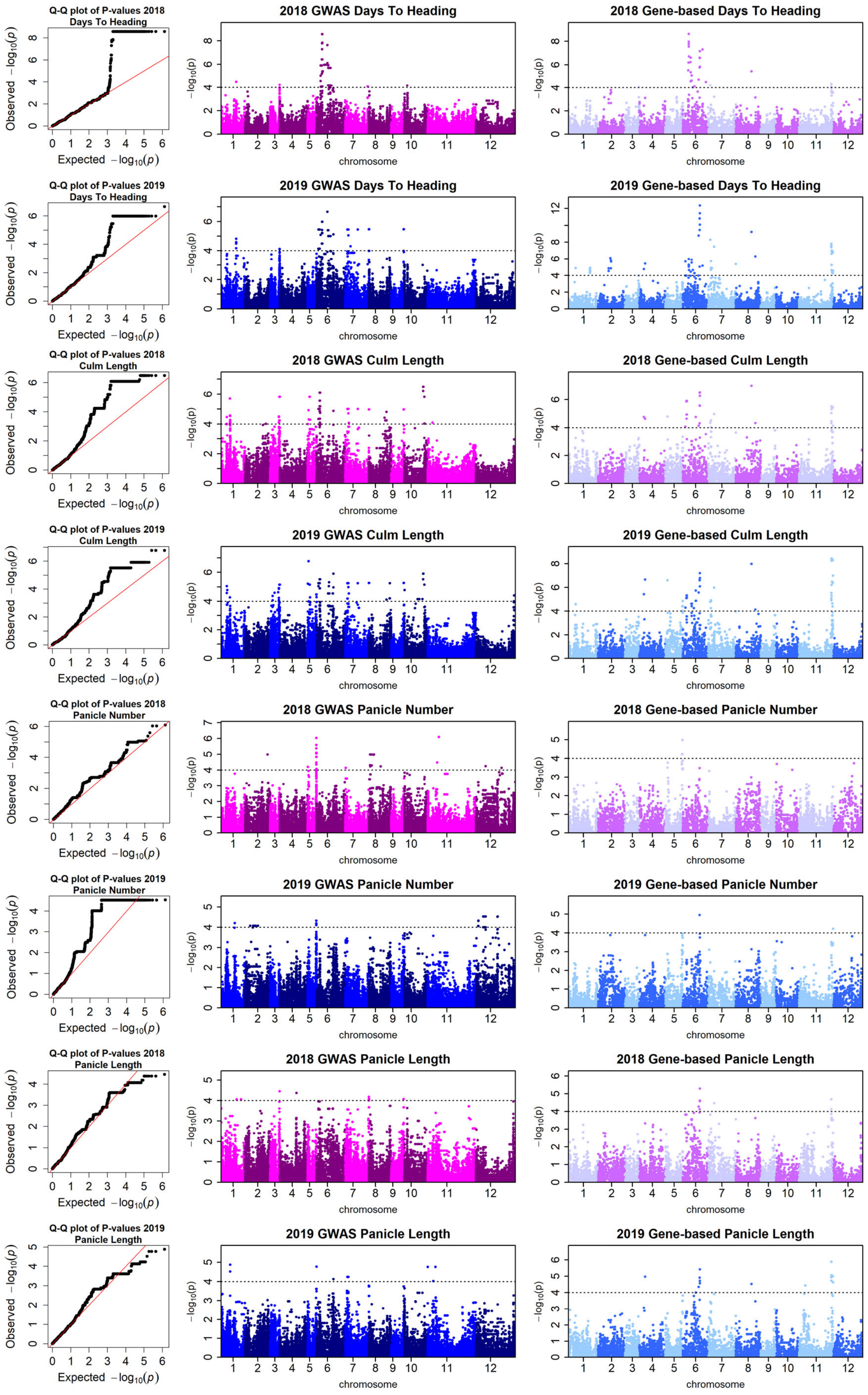

Figure S4. Manhattan plots of GWAS, gene-based association study, and their quantile-quantile plots. The names of the traits are shown at the top of each graph.

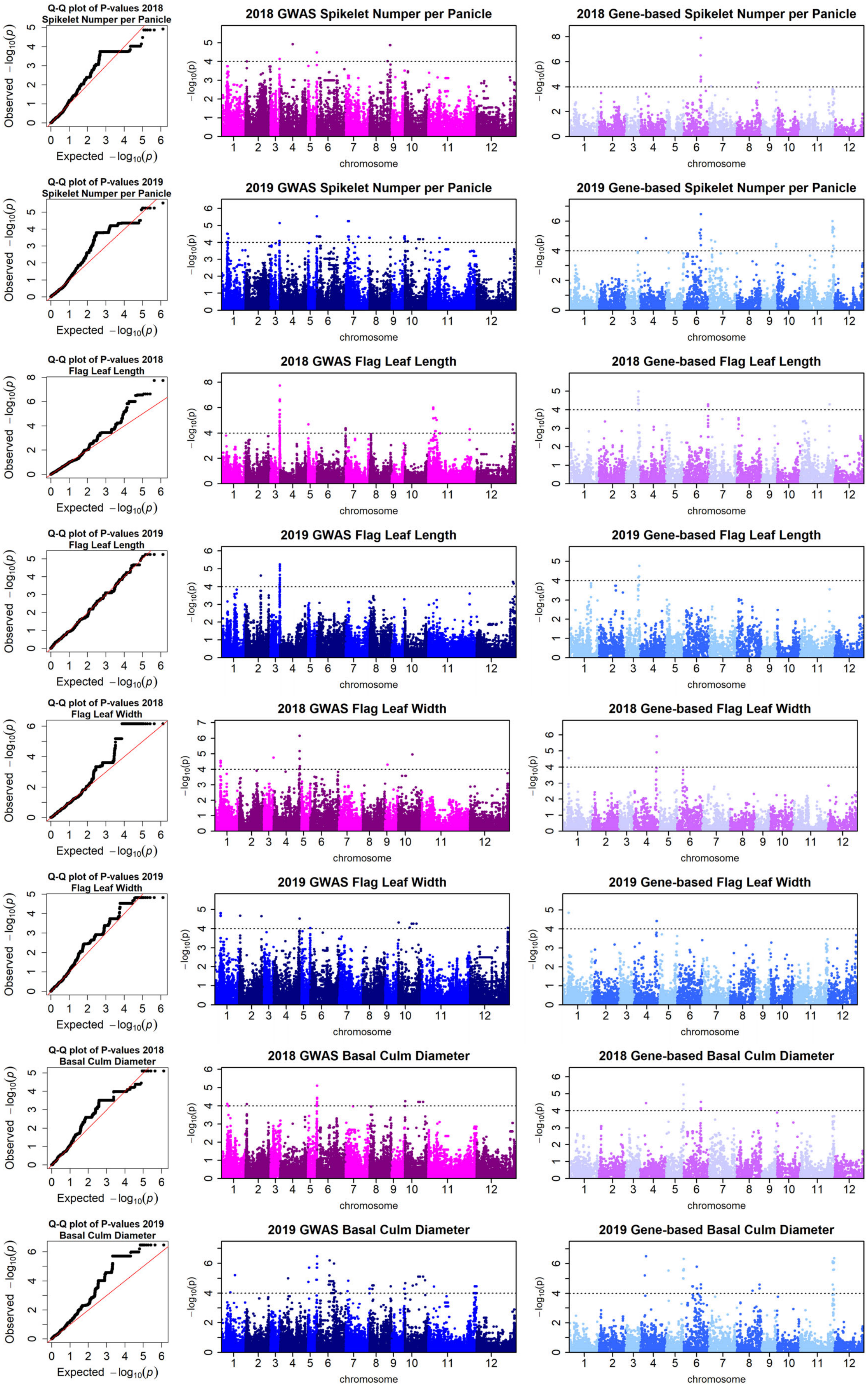

Figure S4. (Continued)

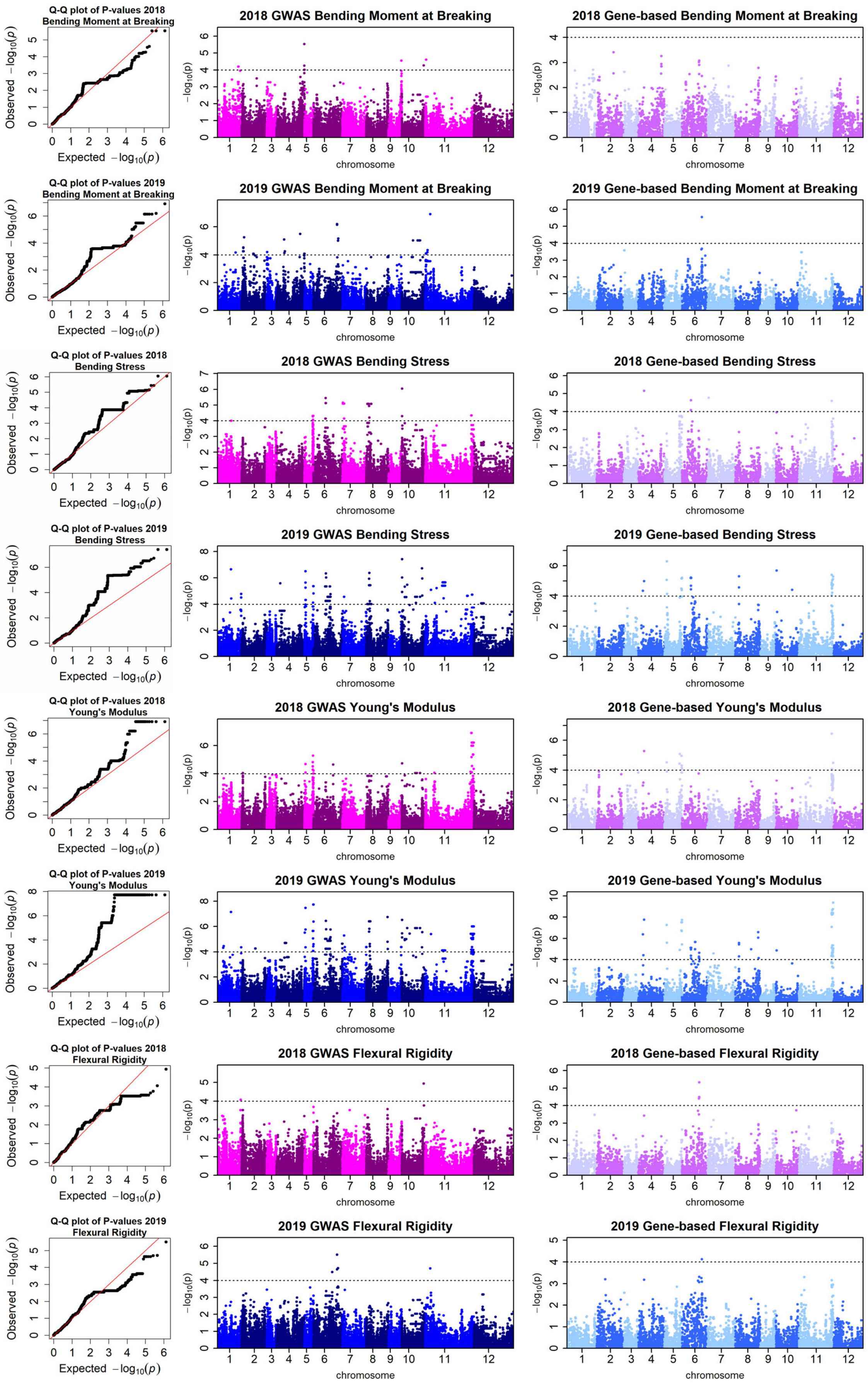

Figure S4. (Continued)

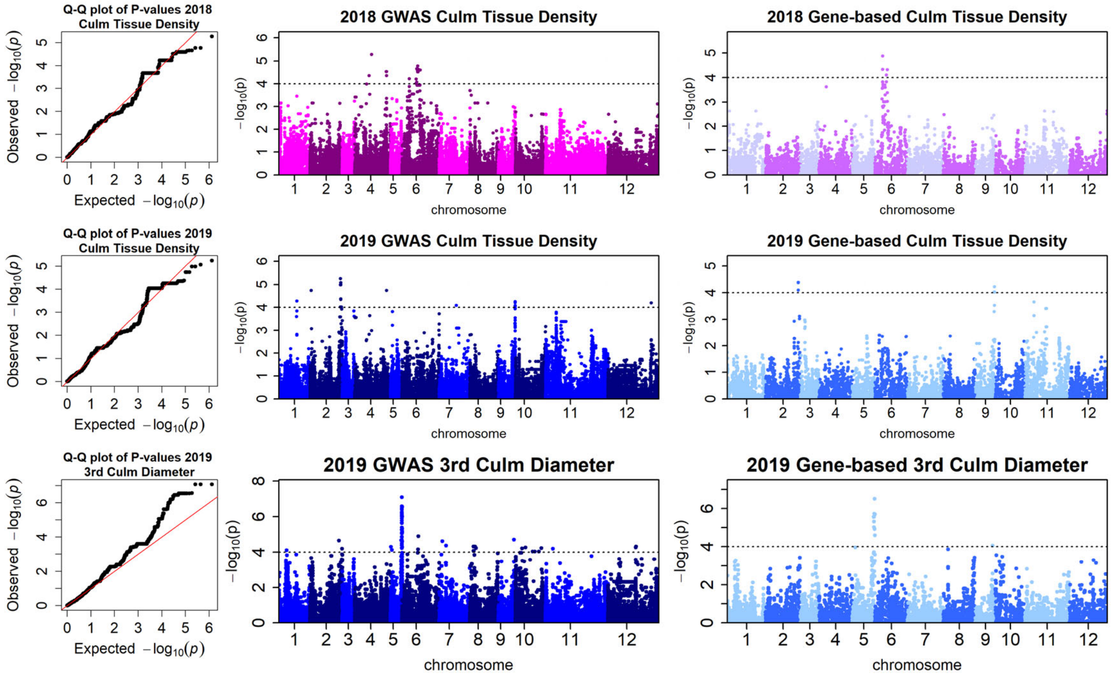

Figure S4. (Continued)

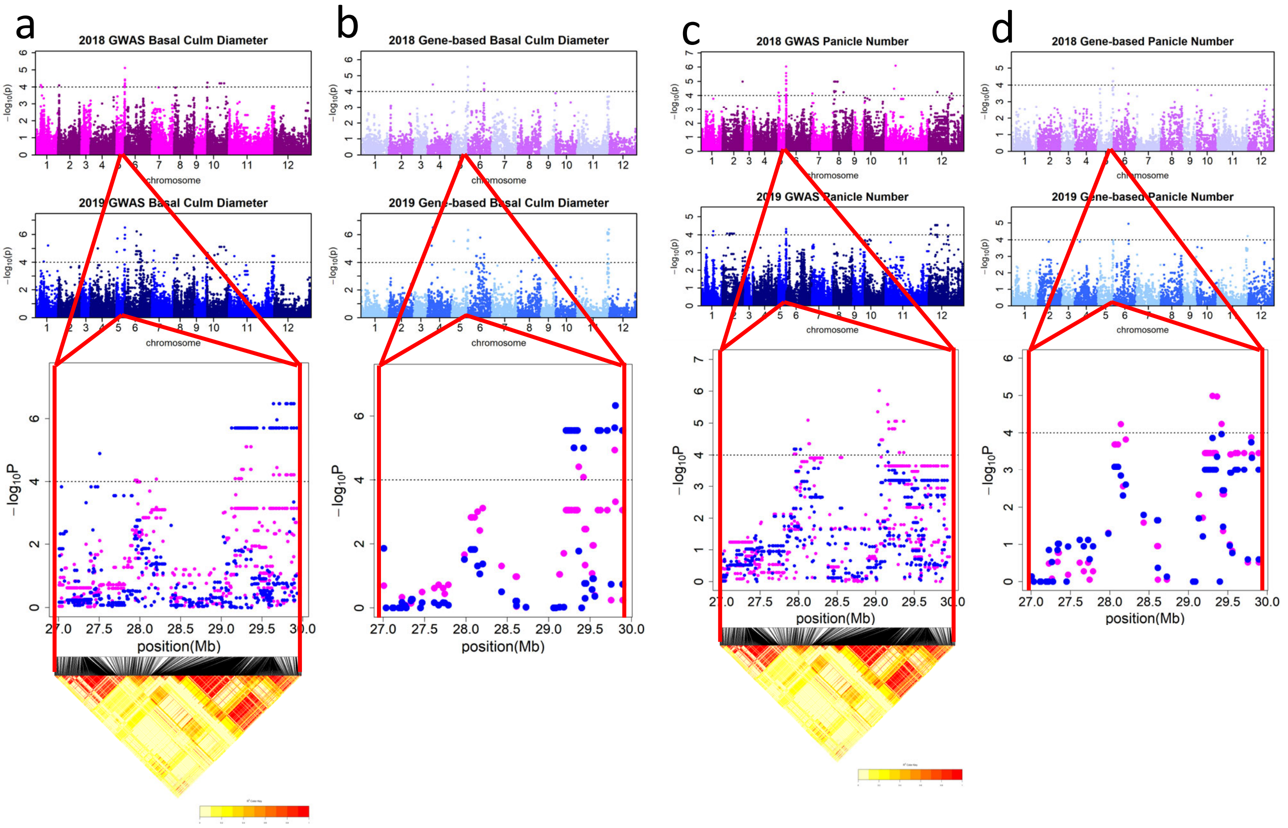

Figure S5. The candidate region detected between 27 Mb and the terminal end of chromosome 5.

The figure below the Manhattan plots is an enlarged view of the candidate region. Red and blue plots indicate the results of 2018 and 2019. (a) The Manhattan plot of GWAS for basal culm diameter. (b) The Manhattan plot of the gene-based association study for basal culm diameter. (c) The Manhattan plot of GWAS for panicle number. (d) The Manhattan plot of the gene-based association study for panicle number.

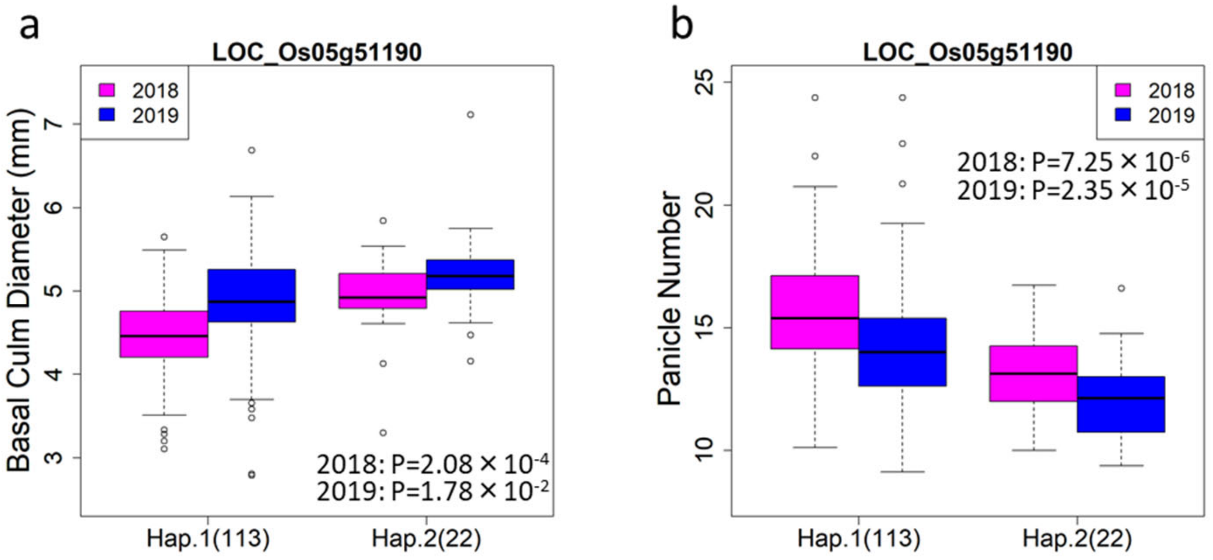

Figure S6. Variances of traits for each haplotype of *LOC_Os05g51190*.
(a) The basal culm diameter (b) panicle number. Differences between the haplotypes were analyzed by Welch's t-test.

Table S1. List of the 135 cultivars used in this research

Cultivar name	Origin	Registration Year (bred)
KIRARA 397	Hokkaido	1988
HOSHINOYUME	Hokkaido	1996
YUKIHIKARI	Hokkaido	1984
HAYAMASARI	Hokkaido	1988
HATSUSHIZUKU	Hokkaido	1998
YUKARA	Hokkaido	1962
TSUGARUROMAN	Tohoku	1996
YUMEAKARI	Tohoku	1999
MUTSUHOMARE	Tohoku	1986
FUKEI 175	Tohoku	(1993)
AKIHIKARI	Tohoku	1976
REIMEI	Tohoku	1966
FUJISAKA 5	Tohoku	1949
TOYONISHIKI	Tohoku	1969
OUU 197	Tohoku	(1937)
CHIYOHONAMI	Tohoku	1987
HITOMEBORE	Tohoku	1991
MANAMUSUME	Tohoku	1997
AKITAKOMACHI	Tohoku	1984
OKINIIRI	Tohoku	1996
FUKUHIBIKI	Tohoku	1993
HIMENOMOCHI	Tohoku	1972
KINUHIKARI	Hokuriku	1988
DONTOKOI	Hokuriku	1995
ITADAKI	Hokuriku	2000
NORIN 1	Hokuriku	1931
HONEN-WASE	Hokuriku	1955
TODOROKI-WASE	Hokuriku	1968
KOSHIHIKARI	Hokuriku	1956
HANAECHIZEN	Hokuriku	1991
YUKINOSEI	Hokuriku	1989
GOHYAKUMANGOKU	Hokuriku	1957
KOGANEMOCHI	Hokuriku	1956
KOSHIJI-WASE	Hokuriku	1953
GOROPIKARI	Kanto	1994
FUSAOTOME	Kanto	1997
HATSUSHIMO	Tokai	1950
AICHINOKAORI	Tokai	1987
MATSURIBARE	Tokai	1994
ASANOHIKARI	Tokai	1987
TSUKINOHIKARI	Tokai	1985
DAICHINOKAZE	Tokai	1999
MINEASAHI	Tokai	1980
NORIN 29	Tokai	1945
AKEBONO	Tokai	1953
NIHONMASARI	Kanto	1973
MILLENISHIKI	Kanto	2000
SATOJIMAN	Kanto	2005
OTOMEMOCHI	Tohoku	1966
YAMABIKO	Tokai	1958
NAKATE-SHINSENBON	Tokai	1950
KINMAZE	Tokai	1948
YAMADANISHIKI	Kinki	1936
NORIN 22	Kinki	1943
ASAHI	Landrace	Unknown
REIHO	Kyusyu	1969
HIYOKUMOCHI	Kyusyu	1971
HOUYOKU	Kyusyu	1961
JIKKOKU	Landrace	Unknown
NORIN 18	Kyusyu	1941
HINOHIKARI	Kyusyu	1989
NISHIHOMARE	Kyusyu	1979
KOGANEMASARI	Kyusyu	1976
YUMETSUKUSI	Kyusyu	1994
AIKOKU	Landrace	Unknown
ASAHI	Landrace	Unknown
OBA	Landrace	Unknown
KAMEJI	Landrace	Unknown
KAMENOO	Landrace	Unknown
SHINRIKI	Landrace	Unknown
TAKENARI	Landrace	Unknown
FUTABA	Tokai	1938
NIPPONBARE	Tokai	1963
KIHOU	Tokai	1968
KOGANEBARE	Tokai	1980
TAICHUNG 65	Taiwan	1927
EIKO	Hokkaido	1942
AKAGE	Landrace	Unknown
FUJIHIKARI	Chugoku	1977
BENISENGOKU	Kyusyu	1953
MORINOKUMASAN	Kyusyu	1996
YUMEHITACHI	Kanto	1997
ASAHINOYUME	Tokai	1999
AKANEZORA	Tokai	1991
HATSUBOSHI	Tokai	1977
CHIYONISHIKI	Tokai	1985
MENKOINA	Tohoku	1999
NOTOHIKARI	Hokuriku	1985
HOHOHONOHO	Hokuriku	1993
YUMEHIKARI	Kyusyu	1990
NATSUHIKARI	Kyusyu	1983
HAENUKI	Tohoku	1992
DOMANNAKA	Tohoku	1992
AKINISHIKI	Hokuriku	1974
HARURU	Chugoku	1997
NORIN 6	Kinki	1936
NORIN 8	Kinki	1937
MORITA-WASE	Landrace	Unknown
RIKUU 132	Tohoku	1921
JYOSYU	Landrace	Unknown
SENICHI	Landrace	Unknown
GINBOUZU	Landrace	Unknown
RIKUU 20	Landrace	Unknown
KAMENOO 4	Landrace	Unknown
OMACHI	Landrace	Unknown
SEKITORI	Landrace	Unknown
BOZU	Landrace	Unknown
SHIROSENBON	Landrace	Unknown
HATSUNISHIKI	Tohoku	1954
YAMASENISIKI	Tohoku	1962
SASASHIGURE	Tohoku	1952
SASANISHIKI	Tohoku	1963
YUKIMARU	Hokkaido	1993
NANATSUBOSHI	Hokkaido	2001
TSUYAHIME	Tohoku	2009
OBOROZUKI	Hokkaido	2003
YUMEPIRIKA	Hokkaido	2008
HOSHIMARU	Hokkaido	2006
DAICHINOHOSHI	Hokkaido	2003
MOEMINORI	Tohoku	2006
AKIDAWARA	Kanto	2009
YAMADAWARA	Kanto	2011
MINEAHARUKA	Tokai	2007
MIZUHONOKAGAYAKI	Hokuriku	2008
EMINOKIZUNA	Hokuriku	2011
AKISAKARI	Hokuriku	2008
KOINOYOKAN	Chugoku	2014
HIMEGONOMI	Chugoku	2010
HAIGOKORO	Chugoku	2012
OIDEMAI	Shikoku	2011
NANGOKUSODACHI	Shikoku	2005
KINUMUSUME	Kyusyu	2005
NIKOMARU	Kyusyu	2005
TACHIHARUKA	Kyusyu	2012
NATSUHONOKA	Kyusyu	2015

Table S2. Candidate genes of *qBCD5-2* and their information.

MSU_ID	IRGSP_ID	Start	End	Note	Oryzabase Symbol	Oryzabase Name
LOC_Os05g50900	*Os05g0586300*	29208375	29210085	Similar to FMA (FAMA)%3B DNA binding / transcription activator/ transcription factor. (Os05t0586300-00)	OsbHLH051	basic helix-loop-helix protein 051
LOC_Os05g50930	*Os05g0586600*	29225038	29227592	Similar to SIGE (RNA polymerase sigma subunit E)%3B DNA binding / DNA-directed RNA polymerase/ sigma factor/ transcription factor. (Os05t0586600-01)	OsSIG5%2C OsSig5%2C Os Sig5	Sigma factor SIG5%2C SIG5 homolog
LOC_Os05g50960	*Os05g0587000*	29240182	29242422	Pectin lyase fold/virulence factor domain containing protein. (Os05t0587000-01)	OsPGL26%2C PGL26	Polygalacturonases-Like 26%2C PG-like 26
LOC_Os05g50970	*Os05g0587100*	29244069	29249086	Protein phosphatase 2C family protein. (Os05t0587100-01)	OsPP2C52%2C PP2C52%2C OsPP80	protein phosphatase 2C52%2C protein phosphatase 2C 52%2C protein phosphatase 80
LOC_Os05g50980	*Os05g0587200*	29251590	29255848	Conserved hypothetical protein. (Os05t0587200-01)	OsSET23	SET protein 23
LOC_Os05g51010	-	-	-	-	-	-
LOC_Os05g51070	*Os05g0588250*	29308010	29308759	Hypothetical protein. (Os05t0588250-00)	OsJ_RLL1	
LOC_Os05g51080	-	-	-	-	-	-
LOC_Os05g51100	*Os05g0588600*	29313924	29314369	Hypothetical gene. (Os05t0588550-01)	-	-
LOC_Os05g51110	*Os05g0588700*	29317649	29324873	Paired amphipathic helix domain containing protein. (Os05t0588700-01)	-	-
LOC_Os05g51140	*Os05g0589000*	29336113	29340355	Uncharacterised protein family UPF0121 domain containing protein. (Os05t0589000-01)%3BUncharacterised protein family UPF0121 domain containing protein. (Os05t0589000-02)	-	-
LOC_Os05g51150	*Os05g0589200*	29341476	29344660	RNA polymerase sigma factor%2C region 2 domain containing protein. (Os05t0589200-01)	OsSIG3	Sigma factor SIG3
LOC_Os05g51160	*Os05g0589400*	29347252	29348581	Similar to I-box binding factor (Fragment). (Os05t0589400-01)%3BSimilar to I-box binding factor (Fragment). (Os05t0589400-02)	R2R3-MYB	MYB-related transcription factor
LOC_Os05g51190	*Os05g0589700*	29359930	29364139	Similar to cDNA%2C clone: J090072P03%2C full insert sequence. (Os05t0589700-01)	OsRLCK191	Receptor-like Cytoplasmic Kinase 191
LOC_Os05g51290	-	-	-	-	-	-
LOC_Os05g51620	*Os05g0594500*	29595609	29601356	Invasin/intimin cell-adhesion domain containing protein. (Os05t0594500-01)%3BHypothetical conserved gene. (Os05t0594500-02)	-	-
LOC_Os05g51660	*Os05g0595000*	29625036	29625780	Allergen V5/Tpx-1 related family protein. (Os05t0595000-01)	OsPR1#051%2C OsPR1-51	pathogenesis-related protein 1-51%2C PR protein 1-51
LOC_Os05g51770	-	-	-	-	-	-
LOC_Os05g51920	-	-	-	-	-	-
LOC_Os05g51930	-	-	-	-	-	-
LOC_Os05g52060	-	-	-	-	-	-

Table S3. Candidate genes of *q3CD5* and their information.

MSU_ID	IRGSP_ID	Start	End	Note	Oryzabase Symbol	Oryzabase Name
LOC_Os05g48910	-	-	-	-	-	-
LOC_Os05g48940	*Os05g0564000*	28073023	28076506	Conserved hypothetical protein. (Os05t0564000-00)	-	-
LOC_Os05g48990	*Os05g0564500*	28100295	28105340	WUSCHEL-related homeobox transcription factor (WOX9) homolog%2C Regulation of the uniform growth of shoots (Os05t0564500-01)	OsWOX9C%2C DWL2	WUSCHEL-related homeobox 9C%2C DWT-LIKE 2
LOC_Os05g49090	-	-	-	-	-	-
LOC_Os05g49150	*Os05g0566500*	28202128	28204227	Similar to Initiation factor 3d (Fragment). (Os05t0566500-01)%3BSimilar to Initiation factor 3d (Fragment). (Os05t0566500-02)	eIF-3d%2C OseIF3d	"eukaryotic translation initiation factor 3%2C subunit d"%2C Eukaryotic translation initiation factor 3 subunit d
LOC_Os05g51070	*Os05g0588250*	29308010	29308759	Hypothetical protein. (Os05t0588250-00)	OsJ_RLL1	
LOC_Os05g51190	*Os05g0589700*	29359930	29364139	Similar to cDNA%2C clone: J090072P03%2C full insert sequence. (Os05t0589700-01)	OsRLCK191	Receptor-like Cytoplasmic Kinase 191
LOC_Os05g51290	-	-	-	-	-	-
LOC_Os05g51500	*Os05g0592600*	29528780	29534681	Translation initiation factor 2 related domain containing protein. (Os05t0592600-01)%3BHypothetical conserved gene. (Os05t0592600-02)%3BHypothetical conserved gene. (Os05t0592600-03)	-	-
LOC_Os05g51510	*Os05g0592800*	29538446	29542019	Splicing variant of protein phosphatase 2C 53 (Os05t0592800-01)%3BClade A type 2C protein phosphatase%2C ABA signaling%2C Regulation of root development%2C Drought resistance%2C Stomatal closure (Os05t0592800-02)%3BSimilar to Protein phosphatase 2C ABI2 (EC 3.1.3.16) (PP2C) (Abscisic acid- insensitive 2). (Os05t0592800-03)	OsPP2C53%2C PP2C53%2C OsPP81%2C OsABIL2%2C ABIL2	protein phosphatase 2C53%2C protein phosphatase 2C 53%2C protein phosphatase 81%2C OsABI-LIKE2%2C ABI-LIKE2%2C ABI-LIKE 2
LOC_Os05g51920	-	-	-	-	-	-

Table S4. Cultivars belonging to two haplotypes on *LOC_Os05g51190* .

Hap. 1 (113)

Cultivar name	Registration Year (bred)
KIRARA 397	1988
HOSHINOYUME	1996
YUKIHIKARI	1984
HAYAMASARI	1988
HATSUSHIZUKU	1998
YUKARA	1962
TSUGARUROMAN	1996
YUMEAKARI	1999
FUKEI 175	(1993)
TOYONISHIKI	1969
CHIYOHONAMI	1987
HITOMEBORE	1991
MANAMUSUME	1997
AKITAKOMACHI	1984
OKINIIRI	1996
KINUHIKARI	1988
DONTOKOI	1995
ITADAKI	2000
NORIN 1	1931
HONEN-WASE	1955
TODOROKI-WASE	1968
KOSHIHIKARI	1956
HANAECHIZEN	1991
YUKINOSEI	1989
KOSHIJI-WASE	1953
GOROPIKARI	1994
FUSAOTOME	1997
HATSUSHIMO	1950
AICHINOKAORI	1987
MATSURIBARE	1994
ASANOHIKARI	1987
TSUKINOHIKARI	1985
DAICHINOKAZE	1999
MINEASAHI	1980
NORIN 29	1945
AKEBONO	1953
NIHONMASARI	1973
MILLENISHIKI	2000
SATOJIMAN	2005
YAMABIKO	1958
NAKATE-SHINSENBON	1950
KINMAZE	1948
YAMADANISHIKI	1936
NORIN 22	1943
ASAHI	Unknown
REIHO	1969
HIYOKUMOCHI	1971
HOUYOKU	1961
JIKKOKU	Unknown
NORIN 18	1941
HINOHIKARI	1989
NISHIHOMARE	1979
KOGANEMASARI	1976
YUMETSUKUSI	1994
ASAHI	Unknown
OBA	Unknown
KAMEJI	Unknown

Cultivar name	Registration Year (bred)
KAMENOO	Unknown
SHINRIKI	Unknown
TAKENARI	Unknown
FUTABA	1938
NIPPONBARE	1963
KIHOU	1968
KOGANEBARE	1980
FUJIHIKARI	1977
BENISENGOKU	1953
MORINOKUMASAN	1996
YUMEHITACHI	1997
ASAHINOYUME	1999
AKANEZORA	1991
HATSUBOSHI	1977
CHIYONISHIKI	1985
MENKOINA	1999
NOTOHIKARI	1985
HOHOHONOHO	1993
YUMEHIKARI	1990
NATSUHIKARI	1983
HAENUKI	1992
DOMANNAKA	1992
AKINISHIKI	1974
HARURU	1997
NORIN 8	1937
JYOSYU	Unknown
GINBOUZU	Unknown
SEKITORI	Unknown
SHIROSENBON	Unknown
HATSUNISHIKI	1954
YAMASENISIKI	1962
SASASHIGURE	1952
SASANISHIKI	1963
YUKIMARU	1993
NANATSUBOSHI	2001
TSUYAHIME	2009
OBOROZUKI	2003
YUMEPIRIKA	2008
HOSHIMARU	2006
DAICHINOHOSHI	2003
MOEMINORI	2006
AKIDAWARA	2009
YAMADAWARA	2011
MINEAHARUKA	2007
MIZUHONOKAGAYAKI	2008
EMINOKIZUNA	2011
AKISAKARI	2008
KOINOYOKAN	2014
HIMEGONOMI	2010
HAIGOKORO	2012
OIDEMAI	2011
NANGOKUSODACHI	2005
KINUMUSUME	2005
NIKOMARU	2005
TACHIHARUKA	2012
NATSUHONOKA	2015

Hap. 2 (22)

Cultivar name	Registration Year (bred)
MUTSUHOMARE	1986
AKIHIKARI	1976
REIMEI	1966
FUJISAKA 5	1949
OUU 197	(1937)
FUKUHIBIKI	1993
HIMENOMOCHI	1972
GOHYAKUMANGOKU	1957
KOGANEMOCHI	1956
OTOMEMOCHI	1966
AIKOKU	Unknown
TAICHUNG 65	1927
EIKO	1942
AKAGE	Unknown
NORIN 6	1936
MORITA-WASE	Unknown
RIKUU 132	1921
SENICHI	Unknown
RIKUU 20	Unknown
KAMENOO 4	Unknown
OMACHI	Unknown
BOZU	Unknown
